# Motor imagery classification method based on relative wavelet packet entropy brain network and improved lasso

**DOI:** 10.3389/fnins.2023.1113593

**Published:** 2023-02-03

**Authors:** Manqing Wang, Hui Zhou, Xin Li, Siyu Chen, Dongrui Gao, Yongqing Zhang

**Affiliations:** ^1^School of Life Sciences and Technology, University of Electronic Science and Technology of China, Chengdu, China; ^2^School of Computer Science, Chengdu University of Information Technology, Chengdu, China

**Keywords:** motor imagery, brain function network, lasso, relief-f, brain-computer interface

## Abstract

Motor imagery (MI) electroencephalogram (EEG) signals have a low signal-to-noise ratio, which brings challenges in feature extraction and feature selection with high classification accuracy. In this study, we proposed an approach that combined an improved lasso with relief-f to extract the wavelet packet entropy features and the topological features of the brain function network. For signal denoising and channel filtering, raw MI EEG was filtered based on an R^2^ map, and then the wavelet soft threshold and one-to-one multi-class score common spatial pattern algorithms were used. Subsequently, the relative wavelet packet entropy and corresponding topological features of the brain network were extracted. After feature fusion, mutcorLasso and the relief-f method were applied for feature selection, followed by three classifiers and an ensemble classifier, respectively. The experiments were conducted on two public EEG datasets (BCI Competition III dataset IIIa and BCI Competition IV dataset IIa) to verify this proposed method. The results showed that the brain network topology features and feature selection methods can retain the information of EEG more effectively and reduce the computational complexity, and the average classification accuracy for both public datasets was above 90%; hence, this algorithms is suitable in MI-BCI and has potential applications in rehabilitation and other fields.

## 1. Introduction

As a new interactive technology, brain-computer interface (BCI) combines biomedical and computer fields to establish a connection between human brain and computer, and continuously expand its application in recent years (Zhao et al., 2020). Among various BCI systems, motor imagery (MI) BCI collects the brain electrical signals during imaginary limb movements of subjects, which is proposed as a candidate approach in motor skill learning and medical rehabilitation (Bigirimana et al., 2020). However, compared with other BCI systems such as P300 and steady-state visual-evoked potential BCI, MI BCI presents a poor performance (Park et al., 2021).

Previous classification tasks of motor imagery primarily focused on improving the feature extraction algorithm. Owing to the characteristics of electroencephalogram (EEG) signals, the common spatial pattern (CSP) algorithm is often used to extract features in the spatial domain (Sharma et al., 2018). In 2018, David et al. proposed a regularized CSP method based on frequency bands and sorted the mutual information between the frequency bands to extract the features. Then they calculated the distance between the feature and label using the second normal form, and performed classification with the nearest neighbor (Park et al., 2018). Zhang et al. proposed a CSP algorithm that optimized both the filter band and the time window to extract features, and an accuracy rate of 88.5% was achieved on the BCI public four-category dataset with a support vector machine (SVM) classification (Jiang et al., 2020). In 2018, Vasilisa proposed a feature weighting and regularization method to optimize the current CSP method to avoid loss of feature information. After the minimum Mahalanobis distance classification, the accuracy of the four-class dataset reached 88.6% (Mishuhina and Jiang, 2018). These mentioned improved CSP algorithm overcomes some of the problems of the traditional CSP algorithm, it still exhibits certain shortcomings, such as it is unsuitable for processing multiclass EEG data.

In addition to feature extraction, studies have been made to improve the performance of feature selection and classification algorithm. In Udhaya Kumar and Hannah Inbarani (2017), the particle swarm optimization (PSO) algorithm combined with a rough set was used to retain features which contribute to the classification accuracy. With the neighborhood rough set classifier, the final average classification accuracy rate in the IIa dataset in BCI competition IV reached 73.1%. In Selim et al. (2018) selected the most distinctive CSP features and optimized SVM parameters by applying a hybrid attractor metagene algorithm and a bat optimization algorithm, and obtained an average classification accuracy rate of 78.3% in the same dataset as that mentioned above (Chu et al., 2018). At this stage, owing to the rapid development of the Riemannian geometry, researchers have used the Riemann minimum distance for pattern classification of EEG signals. In 2019, Javier proposed an improved contraction covariance matrix to handle small sample data more effectively, and subsequently processed the IIa dataset through the Riemann minimum mean distance classifier, and the average classification accuracy rate reached 79.6% (Olias et al., 2019). However, some problems persisted in Riemannian approaches, for example, as the number of the dimension of the covariance matrix rises, the worst the accuracy become (Yger et al., 2017).

To improve the accuracy of feature classification, a new algorithm model based on improved lasso and relief-F was designed in this study. During feature extraction, the relative wavelet packet energy entropy feature of the EEG signal, as well as the variance and mean of the multiclass score common spatial pattern (mSCSP) were extracted. These three features can not only effectively extract the time-frequency-spatial domain information of the signal, but also are suitable for analyzing biological non-stationary signals. Subsequently, feature fusion was performed on the obtained features to overcome the problem of low classification accuracy caused by a single feature. To address the redundancy and high computational complexity of fusion features, a feature selection method based on mutcorLasso and the relief-F algorithm was proposed to retain important features and eliminate redundant ones. Finally, four different classifiers were used to verify the effect of classification, including the K nearest neighbor (KNN), contraction linear discriminant analysis (sLDA), random forest (RF), and an ensemble classifier (Ensemble).

## 2. Materials and methods

**Figure 1:**
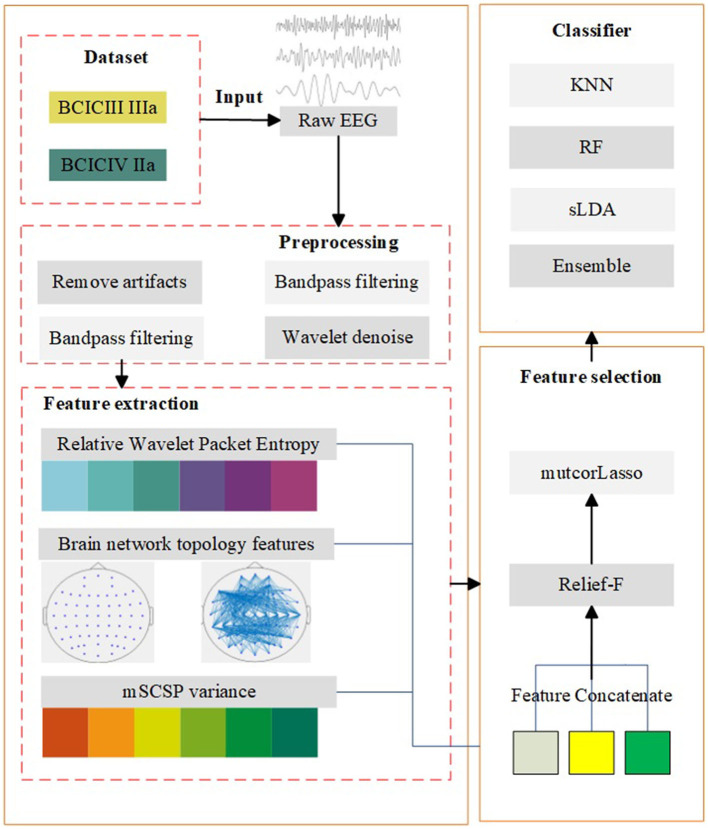
Algorithm model. The diagram consists of five main parts: data introduction, preprocessing, feature extraction, feature selection, and classification.

In order to improve the MI EEG classification accuracy, a recognition method based on brain network and improved lasso was proposed in this paper. A flowchart of the proposed model is shown in Figure 1, which includes data introduction, preprocessing, feature extraction, feature selection, and classification. The feature extraction algorithm mentioned is based on the brain network model framework. The edge weight is set according to the relative wavelet packet entropy, and the threshold selection is based on the global network sparsity when the brain network is constructed. In addition, a feature selection method based on lasso method and presents some improvements to the traditional lasso was proposed. The mutual information and correlation between features are considered for the construction of the objective function of lasso, and then the relief-f algorithm is added for further feature selection.

### 2.1. Data preprocessing

The first step of preprocessing is to remove bad channels with low signal-to-noise ratio by interpolation or average. The next step is band-pass filtering which significantly affect the classification performance of EEG. In this study, the *R*^2^ map is calculated using the power spectral density (PSD) to obtain the frequency band that contains the largest amount of information for each dataset (Choi et al., 2020). In addition, because the signal-to-noise ratio of EEG is extremely low, the data must be denoised and the wavelet soft threshold method was used to perform denoising. The above three steps are serial processing to avoid confusion caused by the entanglement of Midway data.

#### 2.1.1. Wavelet soft threshold denoising algorithm

When the EEG signal undergoes a wavelet decomposition, the amplitude of the wavelet coefficients of EEG is greater than the noise. The noisy signal is decomposed by the orthogonal wavelet base at various scales at a low resolution (Khoshnevis and Sankar, 2020). For the decomposition value at high resolution, the wavelet coefficients whose amplitude is below the threshold were set to zero, and the wavelet coefficients above the threshold are reduced correspondingly or directly retained. Finally, the wavelet coefficients obtained after processing are reconstructed using the inverse wavelet technique, and the denoised EEG is restored.

#### 2.1.2. Multi-score common spatial pattern

The spatial filtering technique is suitable for processing the multidimensional signals, such as EEG (Park et al., 2014). This algorithm mainly improves the CSP algorithm to select EEG channels. By calculating the score of the projection matrix for all the channels, the channel with the highest score for each class is selected and combined to obtain the optimal filter channels. The algorithm not only maximizes the variance difference between classes but also reduces the cost of computing resources.

### 2.2. Feature extraction

In this paper, the wavelet packet method is used to extract the detail and approximate coefficients of EEG. The energy entropy values of these coefficients are calculated, and a brain function network based on these energy entropy values is constructed to extract the topological features. Because the mSCSP algorithm in the previous step amplifies the variance of different samples, the variance characteristics of each sample are also extracted. Finally, the three parts of features are fused to obtain a higher-dimensional matrix. However, the dimensions of the features extracted by the above three different feature extraction algorithms are different, resulting in the situation that some features with large dimensions may have a great impact on the screening results in the subsequent feature screening. Therefore, the feature matrix is standardized and the features with different dimensions are compressed to the range of [0,1] for subsequent processing. The two main feature extraction methods used in this study are as follows.

#### 2.2.1. Relative wavelet packet entropy

Currently, relative wavelet packet entropy has been widely used in processing EEG data. It can efficiently extract the time–frequency domain information, and the low frequency of EEG can be reduced by wavelet packet decomposition technology. Meanwhile, the high-frequency information are extracted to reflect the time–frequency domain information of this part of the EEG signal more effectively. This wavelet packet decomposition method has no redundancy and omissions, therefore, it can perform an efficient time–frequency localization analysis on EEG that contain a large amount of medium and high-frequency information.

In this study, the EEG signal is decomposed into three layers. Therefore, the approximate and detail coefficients of the three layers are obtained, which are *A*_*j*_, *j* = 1, 2, 3 and *D*_*j*_, *j* = 1, 2, 3 where *j* represents the number of decomposition layers. The formula for calculating the energy coefficient of each layer was as follows:

(1) $$\begin{array}{l} \begin{array}{c} E_{j}=\left(A_{j}\left(k\right)+\underset{k}{\sum }D_{j}{\left(k\right)}^{2}\right)/3 \end{array} \end{array}$$

where *k* represents the k-th channel, the approximation coefficients *A*_*j*_ are averaged, and the detail coefficients *D*_*j*_ are used in the second norm. Therefore, both the detail coefficients and approximation coefficients are considered as the energy value of each layer.

Furthermore, because the approximation coefficient is more important in the analysis of EEG signals, the original value of the approximation coefficient is directly used, whereas the detail coefficient is used as part of the energy coefficient. The formula to calculate the total energy is as follows:

(2) $$\begin{array}{l} \begin{array}{c} E_{t}=\underset{j}{\sum }E_{j}^{2}, \end{array} \end{array}$$

where *E*_*t*_ represents the total wavelet packet energy value (Dimitrakopoulos et al., 2018). The relative wavelet energy value can be obtained from the two formulas above, and the specific formula is as follows:

(3) $$\begin{array}{l} P_{j}=\frac{E_{j}}{E_{t}} \end{array}$$

where $\sum_{j}P_{j}=1$, and the distribution of *P*_*j*_ can be used as an important feature of the EEG time–frequency domain. Next, based on the Shannon entropy theory, the wavelet packet energy entropy was calculated (Li and Zhou, 2016). The specific formula is as follows:

(4) $$\begin{array}{l} S_{m}=-\underset{j}{\sum }\left(P_{j}\ln\left(P_{j}\right)\right), \end{array}$$

where *S*_*m*_ represents the relative wavelet packet energy entropy of channel *m*. Based on the formula, the value between channels can be calculated, which provide a foundation for building a brain function network for each dataset.

#### 2.2.2. Brain network

The method to construct a brain function network can be primarily classified into following four steps:

**Node definition**: Each channel electrode after channel selection is used as a node to construct the brain network.

**Weight calculation**: The weight value of the edge in this experiment is the relative energy entropy of wavelet packet designed in the previous section.

**Threshold definition**: The threshold selection criterion used in this experiment is based on sparsity, which is determined as the 30% sparsity standard to ensure that each node is not an isolated node and that the network complexity is low. This is more suitable for subsequent processing.

**Topological feature extraction**: It is primarily aimed at several typical topological features of the constructed brain network, including the degree of the node, clustering coefficient of the node, global efficiency of the brain network, and characteristics of the first and spectral norms of the brain network. The specific formulas are as follows Lee et al. (2018):

The formula for node degree parameter is as follows:

(5) $$\begin{array}{l} k_{i}=\underset{j\in N}{\sum }\left(R_{ij}\right)+\underset{j\in N}{\sum }\left(R_{ji}\right), \end{array}$$

where *R*_*ij*_ and *R*_*ji*_ indicate the edge from node *i* to node *j* and the edge from node *j* to node *i* exist, respectively. The *N* represents the total set of features extracted from the brain topology network, and *k*_*i*_ represents the degree of node *i*, which is calculated by the sum of the outgoing and incoming paths of the node. After calculating the degree of the node, it can be used to calculate the clustering coefficient of the brain network. The specific formula for the calculation is as follows Kakkos et al. (2019):

(6) $$\begin{array}{l} C=\frac{t}{k_{i}*\left(k_{i}-1\right)} \end{array}$$

where t represents the number of triangles around node *i*. The clustering coefficient can reflect the universality of cluster connections around a single node; therefore, it is often analyzed as a feature of the brain function network (Horn et al., 2014). Another feature is the global efficiency of the brain network, which can reflect the degree of connectivity of the entire brain network. The specific formula used for calculation is as follows:

(7) $$\begin{array}{l} E=\frac{1/\sum_{j\in N,j\neq i}d_{ij}}{N-1}, \end{array}$$

where *d*_*ij*_ represents the shortest distance from node *i* to node *j*. The shortest distance was calculated using Dijkstra's algorithm. The starting point is taken as the center and expand outward layer by layer (breadth first search idea) until it is extended to the end point. The order of increasing length produces the shortest path used in this algorithm. That is, after sorting the path lengths of all visible points each time, this algorithm select the shortest path from the corresponding vertex to the source point. Therefore, this algorithm is more suitable for EEG than prim algorithm or Freud algorithm.

The nodes of brain network are defined by reconstructing different node positions on the electrode cap and the corresponding path is composed of the relative wavelet packet entropy coefficient. Then the threshold is set to determine the sparsity of the brain network construction to avoid high computational complexity and feature redundancy. The topological characteristics of these three parts of the brain network can fit the information of entire brain network.

### 2.3. Feature selection

Owing to the higher dimension of the matrix after feature fusion, a significant amount of computing resources is consumed. Therefore, the lasso method based on mutual information and correlation combined with the relief-f method is used for feature selection. Finally, the feature matrix with smaller dimensions is selected, which could reduce the computational complexity and ensure a higher classification performance. The specific details of these two algorithms are as follows:

#### 2.3.1. mutcorLasso

During data training, hundreds or even thousands of variables are involved. Therefore, there are possibilities of overfitting when the dependent variable of the objective function is measured using several variables. Lasso-based methods can be used to perform filtering more efficiently by eliminating some nonessential variables. Therefore, both discrete and continuous data can be processed. In this paper, we propose a lasso method based on mutual information and correlation, which is an improvement on the traditional lasso algorithm. It considers the mutual information and correlation information of features and labels followed by optimization. We modified the objective function of the traditional lasso algorithm, and objective function proposed is as follows:

(8) $$\begin{array}{l} min\left(∥{\text{y}}^{⊤}-{\text{w}}^{⊤}X{∥}_{2}^{2}+\alpha ∥w{∥}_{1}+\beta {\text{w}}^{⊤}Cw\right), \end{array}$$

where *y* and *X* are formula elements in the traditional Lasso algorithm, *y* represents the label of the dataset, and *X* represents the characteristic matrix calculated according to the least squares method. *C* is the squared mutual information correlation matrix, and *w* is the weight coefficient of each feature vector. α and β are the learning rates that control the optimization speed of the entire objective function. If the setting is extremely small, the local optimal value can be obtained easily; if extremely large, the amplitude of result fluctuates significantly, and the global optimal value can be obtained easily. In this study, the initial value of α is set to 0.5 and β to 0.1, the values are updated to get a better accuracy based on these two parameter combinations. The formula to calculate matrix *C* is as follows:

(9) $$\begin{array}{l} C=R⊙R, \end{array}$$

where *R* is the coefficient matrix of the mutual information and correlation, ⊙ represents the Hadamard product, and the formula to calculate each element in the *R* matrix is as follows:

(10) $$\begin{array}{l} r_{kl}=\frac{\sum_{i=1}^{n}x_{ki}x_{li}}{\sqrt{\sum_{i=1}^{n}x_{ki}^{2}}\sqrt{\sum_{i=1}^{n}x_{li}^{2}}}+|mutInf\left(x_{ki},x_{li}\right)|, \end{array}$$

where *mutInf* represents the mutual information between two feature vectors. Using this formula, each coefficient of matrix *R* is obtained and used as the basis for the optimization of subsequent objective function. After the general feature is selected using the algorithm above, the feature dimension is still large. Therefore, the experiment will be proceeded using the relief-f algorithm, which is typically used at this stage to perform further feature selection.

After feature filtering by the above method, the dimension of feature vector is reduced from 120 to 20, and the relevant redundant features are eliminated.

#### 2.3.2. Relief-f

The basic principle of the algorithm is as follows: first, samples *R* are randomly selected from training set *D* and the *k* nearest neighbor samples *H* are obtained from the same type *R*. Subsequently, the *k* nearest neighbor samples *M* are selected from samples of different types from *R*. Finally, the feature weight is updated using this formula.

In view of the overall dimensions of the dataset and information from relevant studies, we set the *k* nearest neighbor samples to six. To ensure that each sample type is randomly selected, we control the random sampling rate required by the algorithm to be within 30–40%. The distance function is marginally modified, and the distance is set to the absolute value of the difference between elements in two feature vectors, thereby reducing calculation complexity and reflecting the difference between random and selected samples. Finally, the statistics on the *w* value after traversal are obtained, the *w* value of each feature vector is sorted, and feature matrix of lower dimensions is selected. In this algorithm, the update formula of feature weight *w* is as follows:

(11) $$\begin{array}{l} w=w-\sum_{j=1}^{k}diff\left(A,R,H\right)/(mk)\\ \;+\sum_{c\in class(R)}\frac{p(C)}{1-p(class(R))}\frac{\sum_{j=1}^{k}diff\left(A,R,H\right)}{{\left(mk\right)}^{2}} \end{array}$$

where *diff*() represents the difference between the *R* and *H* samples on feature *A*, and *mk* represents the number of total samples. According to the formula, the *w* coefficient can be continuously updated.

After feature filtering by the above method, the dimension of feature vector is reduced from 20 to 10, So it is better suitable for classification tasks with low time complexity.

### 2.4. Classifier

The last component pertains to classification. Four classifiers were used in the experiment, namely KNN, sLDA, RF, and the Ensemble obtained by integrating the three classifiers. These four classifiers can verify whether the proposed algorithm is universal.

KNN is determined by voting the unlabeled samples by the K nearest neighbors (Bablani et al., 2018). sLDA is an improved version of linear discriminant analysis, which is more applicable when the number of training samples is less than the number of features (Tjandrasa and Djanali, 2016). RF is an extension of the traditional decision tree classification algorithm that adds knowledge in the integrated learning field and performs decision classification based on multiple decision trees (Lanata et al., 2020). After verifying the classification accuracy for different number of decision tree on the datasets, we set the number to 10 in the RF. Ensemble is integrated according to the prediction labels finally obtained using the three classifiers mentioned, and it uses the voting method to predict the labels of final ensemble classifier.

The five components above are the specific description of the algorithm model. The following sections focus on the new algorithm proposed herein in feature extraction and feature selection. The pseudo code of the feature selection algorithm above is shown below Table 1.

**Table 1:** The pseudo code of the feature selection algorithm.

**Algorithm: Procedure of mutcorLasso+Relief-f**
**Input**: Feature vector matrix X ∈ *R*^p*n^, corresponding label *y* ∈ *R*^*p*^,
The maximum number of iterations *iter*_*num*_
**mutcorLasso method:**
1: **For** i from 1 to *n* do:
2: **For** j from 1 to *n* do:
3: Calculate mutual information and correlation using Equation (10)
4: **end for**
5: Construct matrix C based on the above coefficients
6: Calculate the Hadamard product following Equation (9)
7: Obtain the fixed matrix B:
8: *B* = *X* ∗ **X**^⊤^ + β ∗ *C*
9: Initialize the *w* coefficient to a random decimal between 0 and 10
10: **While** i < 1,000
11: temp = *w*
12: Calculate diagonal matrix *M*:
13: $M^{\left(t\right)}=diag\left(\sqrt{w_{1}^{\left(t\right)}},\sqrt{w_{2}^{\left(t\right)}},\ldots ,\sqrt{w_{p}^{\left(t\right)}}\right)$
14: Update weight coefficient *w*:
15: $w^{t+1}=M^{t}{\left[M^{t}BM^{t}+\frac{\alpha I_{p}}{2}\right]}^{-1}M^{t}Xy$
16: **if** t > *iter*_*num*_:
17: **break**
18: **end if**
19: According to the value of *w*, the features with *w* of 0 are eliminated
**Relief-f method:**
20: **For** i from 1 to 60:
21: Random select a sample
22: Find 6 neighbor samples from the same class as the sample
23: Find 6 neighbor samples that are different from the sample
24: Update *w* weight using Equation (11)
25: sort w
26: Select the first N-dimensional features according to the value of *w*
**Output**:feature matrix after selecting

### 2.5. Evaluating indicator

The evaluation indicators used in this experiment was accuracy. It is the most important index in the entire classification system and is obtained based on the confusion matrix. The specific formula to calculate it as follows:

(12) $$\begin{array}{l} \begin{array}{c} Accuracy=\frac{True_{num}}{Total_{num}}, \end{array} \end{array}$$

where *True*_*num*_ indicates the number of samples correctly classified, and the *Total*_*num*_ indicates the total number of samples. All the data in the result tables are obtained through 10 fold cross validation.

## 3. Results

### 3.1. Data description

To demonstrate the effectiveness of the proposed method, we conducted the following experiments on the dataset IIIa in BCI competition III (Blankertz et al., 2006) and the dataset IIa in BCI competition IV (Tangermann et al., 2012), as detailed in Table 2.

**Table 2:** Data description.

**Competition**	**Subjects**	**Train**	**Test**
Dataset IIIa, BCI-III	K3b (45 Trials/task)	90	90
	K6b (30 Trials/task)	60	60
	L1b (30 Trials/task)	60	60
Dataset IIa, BCI-IV	A01~A09 (9 Subjects)	144	144

Both datasets include four motor imagery tasks of left hand, right hand, foot or tongue movement.

### 3.2. Experimental parameter settings

#### 3.2.1. Setting of α and β in mutcorLasso algorithm

**Figure 2:**
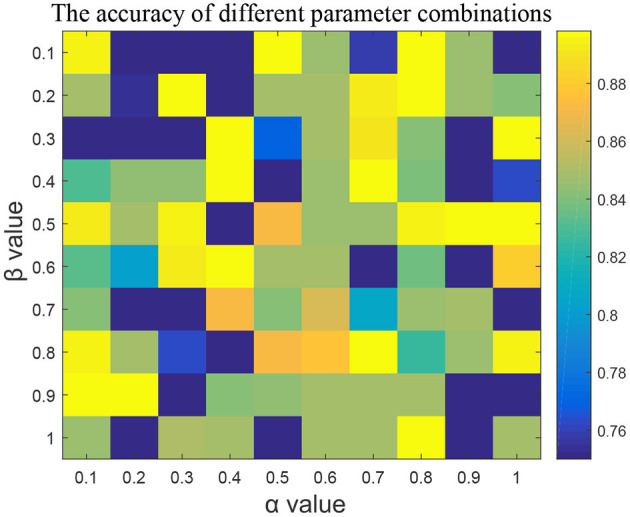
The effect of α and β parameters in mutcorLasso algorithm. As the color is closer to yellow, the higher the accuracy of the classification is.

In the feature selection part, we adjusted two parameters, α and β, used in the algorithm and used five-fold cross-validation to verify the results of RF classifier. Taken K3b dataset as example, Figure 2 represented the learning rates α and β of mutcorLasso algorithm, respectively. Although the parameters range between 0.1 and 1, which is relatively small, it affects the classification accuracy very significantly. It could be seen when α was 0.5 and β was 0.1, the accuracy of the K3b dataset was the best among all these values. Similarly, in the other dataset, the adjacency matrix graph was calculated to reflect a better accuracy based on different parameter combinations in mutcorLasso algorithm, and then the best combination of α and β was determined to improve accuracy.

#### 3.2.2. Setting of bandpass filter parameter

**Figure 3:**
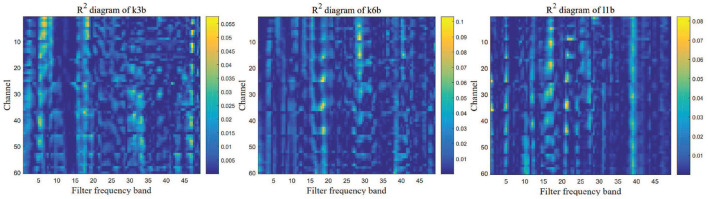
*R*^2^ chart for three EEG datasets. The more information the frequency band contains, the closer is the color to yellow. Therefore, the bandpass filter parameters of each dataset can be determined.

We removed the artifacts from raw EEG data and then calculated the PSD of each sample to construct the *R*^2^ chart that reflects the information of different frequency bands. The three graphs in Figure 3 show the bandpass filter for three datasets, in which the ordinate indicates the number of channels, the x-axis indicates the bandpass filter frequency band, and each square indicates the power of each channel in different filter frequency bands. Based on them, the filter band of the k3b dataset was set to 0.5–20, the k6b was set to 3–30, and the l1b was set to 4–40. Similarly, in the IV2a dataset, bandpass filtering was performed based on the relevant *R*^2^ map to obtain more information.

#### 3.2.3. Setting of wavelet base in wavelet soft threshold denoising

**Figure 4:**
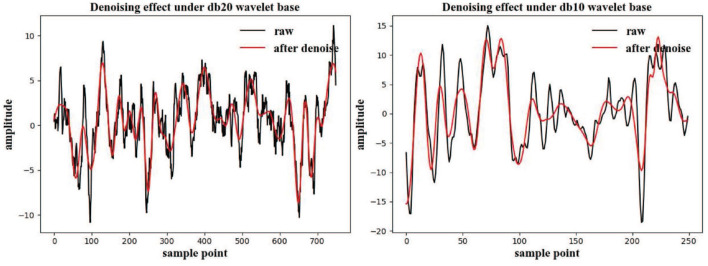
Wavelet soft threshold denoising results under different competition datasets. The black line in the figure represents the original signal, and the red line represents the result after the wavelet-based denoising.

Because of the varied sampling numbers of the two competition datasets, the wavelet bases for these datasets were different. In this experiment, the wavelet soft threshold method was used to perform denoising. It can be inferred from the Figure 4 that the denoised signal can approximately retain the original value of the original signal, and some high-frequency noise signals are directly eliminated. For the 250 samples in the BCI3 IIIa four-class dataset, db10 was selected as the wavelet base, and for the 1,125 samples in the BCI4 IIa four-class dataset, db20 wavelet base was selected (Yang et al., 2018).

#### 3.2.4. Setting of brain network sparsity parameter

**Figure 5:**
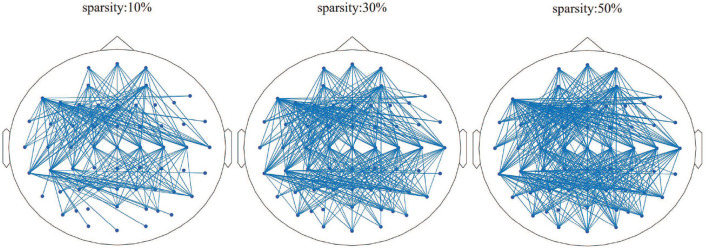
Brain network results in different sparsity situations, as 10, 30, and 50%, respectively.

The brain network parameter selection was verified based on the k3b dataset. After constructing the entire brain network model, we compared the effect of sparsity on the brain network model, as shown in Figure 5. The brain network with the sparsity of 10% contains isolated nodes, which could affect the subsequent extraction of the brain network features. In addition, the brain network with the sparsity of 50% shows an extremely dense overall connection of the brain network, which rises the calculation complexity. Thus, the brain network diagram with the sparsity of 30% will be used to build a brain network and the topological features.

### 3.3. Results of different classifiers on two datasets

#### 3.3.1. Dataset IIIa (BCI-III)

The performance of the algorithm model was verified based on the 50% cross-validation method. This experiment was repeated 20 times and the average accuracy of the entire algorithm model was obtained. In Table 3, the classification accuracy of the existing corresponding algorithms used for Dataset IIIa (BCI-III) is mostly between 80 and 85%, and the accuracy of ensemble classifier exceeded 90% when the training set contained few samples.

**Table 3:** The classification accuracy of different classifiers on dataset IIIa.

**Data**	**sLDA**	**RF**	**KNN**	**Ensemble**
K3b	93.32	89.25	92.62	91.83
K6b	93.71	93.13	91.82	92.19
L1b	92.83	93.19	94.46	93.95

#### 3.3.2. Dataset IIa (BCI-IV)

Training set and test set were used at a ratio of 1:1 to verify the algorithm model on dataset IIa. The experiment was repeated 20 times to obtain the variance value of the entire model. In Table 4, the average accuracy of the nine datasets exceeded 80%, which is better than the optimal average value of 80.9% obtained in the previous paper. In particular, the average accuracy obtained by the RF classifier was approximately 90%, which is a significant improvement. The classification accuracy of the existing corresponding algorithms used for Dataset IIa (BCI-IV) was mostly above 85%, suggesting the algorithm model proposed in this paper can achieve good results on this data set.

**Table 4:** The classification accuracy of different classifiers on dataset IIa.

**Data**	**sLDA**	**RF**	**KNN**	**Ensemble**
A01	86.42	89.03	91.06	90.55
A02	92.35	93.95	92.75	94.26
A03	72.26	88.89	85.74	86.41
A04	64.67	65.86	65.69	65.73
A05	95.81	99.11	97.94	97.94
A06	89.63	99.26	97.62	98.03
A07	90.36	98.33	98.36	97.88
A08	88.98	93.64	92.04	90.25
A09	66.01	77.18	66.35	73.14

### 3.4. Results of fused feature extraction algorithms

In the subsection, the classification accuracy of several feature extraction algorithms mentioned were verified through the five-fold cross-validation method. To avoid the influence of feature selection, the extraction features were directly classified by the RF classifier without feature selection. Table 5 shows that the classification effect of the combination of any two feature extraction methods is better than that of the single feature extraction algorithm alone, and the classification effect obtained by combining the three methods mentioned is the best, approximately 90%. What's more, it's discovered that the variance features obtained by the SCSP facilitated the classification to be the best, followed by the topological features of the brain network.

**Table 5:** The classification accuracy for different feature extraction algorithms.

**Data**	**RWPEE**	**BNTC**	**mSCSP**	**RWPEE+BNTC**	**RWPEE+SCSP**	**BNTC+SCSP**	**Proposed**
A01	56.16	70.16	79.93	76.96	80.51	85.54	**88.65**
A02	61.15	70.83	79.62	76.56	80.62	82.34	**82.64**
A03	63.84	81.94	84.61	82.24	88.91	84.64	**92.52**
A04	63.46	78.22	82.91	78.33	87.92	89.74	**91.96**
A05	64.04	84.86	83.36	84.85	81.81	**85.84**	**85.84**
A06	67.94	80.49	82.95	80.46	82.53	83.26	**93.76**
A07	68.46	73.12	83.46	73.24	82.13	84.04	**93.59**
A08	60.12	78.52	69.69	78.64	78.81	80.25	**82.21**
A09	67.56	74.43	86.38	75.31	82.21	84.65	**94.53**
Mean	63.61	76.91	81.43	78.35	82.86	84.45	**89.55**

RWPEE refers to relative wavelet packet energy entropy, BNTC refers to the brain network topological characteristics of brain network, mSCSP refers to the one-to-one scoring CSP algorithm variance characteristics.

Bold values represent the highest value.

### 3.5. Results of feature extraction algorithms

We compared the proposed feature extraction method with other algorithms, including Sparse Filter Bank Common Spatial Pattern (SFBCSP) (Zhang et al., 2015), Temporally Constrained Sparse Group Spatial Pattern (TSGSP) (Yu et al., 2018) and Discrete Wavelet Decomposition (DWT) (Khatun et al., 2016). We randomly combined features extracted from these four methods with mSCSP variance features, then performed feature selection by the relief algorithm, finally obtained the average accuracy of the mentioned classifiers after a five-fold cross-validation. As shown in the Table 6, in datasets IV2a, the average accuracy exceeds 90% by the proposed feature extraction method combining wavelet packet energy entropy and brain network features. The results suggest that the feature extraction method proposed is better than the other three feature extraction methods.

**Table 6:** The classification accuracy different feature extraction algorithms.

**Data**	**TSGSP**	**SFBCSP**	**DWT**	**Proposed**
A01	86.03	82.73	74.83	**88.63**
A02	74.51	74.22	79.61	**82.63**
A03	91.75	88.55	87.71	**92.51**
A04	75.16	69.56	87.95	**91.95**
A05	81.91	76.34	83.15	**83.66**
A06	69.27	64.64	90.55	**93.78**
A07	89.89	86.88	**94.51**	93.51
A08	**94.48**	90.99	90.31	**92.23**
A09	81.95	72.84	89.91	**94.50**
Mean	82.75	78.55	86.73	**90.33**

Bold values represent the highest value.

### 3.6. Results of feature selection algorithms

Table 7 compared the proposed mutcorLasso method with relief-f, lasso and the combination of these methods. The feature matrix obtained before feature selection is guaranteed to be exactly the same, but different features are adopted in the feature selection part. The selection algorithm controls the features in 20 dimensions to ensure that the feature dimensions selected using different feature selection algorithms are the same. After 50% cross-validation, the proposed algorithm achieved better accuracy of above 90% than other algorithms.

**Table 7:** The classification accuracy for different feature selection algorithms on dataset IIIa.

**Data**	**Relief-f**	**Lasso**	**Lasso+Relief-f**	**Proposed**
K3b	87.84	82.21	89.05	**91.97**
K6b	88.57	82.15	90.25	**92.63**
l1b	86.74	83.33	88.75	**91.55**
Mean	87.75	82.53	89.35	**92.05**

Bold values represent the highest value.

In Table 8, three feature selection algorithms were compared, including Gradient Boosting Decision Tree (GBDT) (Wang et al., 2019), Pearson correlation coefficient (Pearson) (Xu and Deng, 2018) and Particle swarm optimization (PSO) (Wang et al., 2020). These three algorithms are widely used and representative feature selection algorithms of different kinds. According to the results of these datasets, the feature selection algorithm proposed herein yielded better results on seven datasets, with an average accuracy rate of 88.8%, which is an improvement compared with other three feature selection algorithms, 0.8, 4.3, and 4.9%.

**Table 8:** The classification accuracy for feature selection and comparison algorithms on dataset IV2a.

**Data**	**GBDT**	**Pearson**	**Pso+svm**	**Proposed**
A01	91.02	87.71	86.18	**91.91**
A02	84.30	83.42	76.19	**84.83**
A03	90.81	85.61	77.98	**91.52**
A04	**95.22**	88.74	87.41	90.36
A05	88.81	85.91	92.36	**92.75**
A06	**93.92**	90.15	91.68	93.74
A07	92.93	85.76	88.27	**93.49**
A08	85.51	82.88	81.02	**86.48**
A09	69.96	70.44	**74.85**	**74.87**
Mean	88.02	84.54	83.96	**88.85**

Bold values represent the highest value.

## 4. Conclusion

The proposed model effectively integrates seven components: bandpass filtering, wavelet denoising, channel filtering, feature extraction, feature fusion, feature selection, and pattern classification. The main contributions of this study are as follows. Firstly, a complex brain network feature extraction method based on wavelet packet energy entropy was proposed, which not only extracts space–time domain features but also extracts the topological features of the brain network simultaneously, thereby retaining more EEG feature information. Then, a lasso method based on mutual information and correlation was proposed, and the subsequent relief-f algorithm was combined with feature filtering to improve the selected features. The proposed algorithm model can effectively mitigate the problem of low accuracy caused by the scarcity of the training set and achieve precise motion imaging classification. In the future, reducing the computational complexity of the algorithm model and realizing online analysis for a better application in medical rehabilitation will be another research direction of our work.

## Data availability statement

Publicly available datasets were analyzed in this study. This data can be found at: https://www.bbci.de/competition/iii/results/.

## Ethics statement

Ethical review and approval was not required for the study on human participants in accordance with the local legislation and institutional requirements. Written informed consent for participation was not required for this study in accordance with the national legislation and the institutional requirements. Written informed consent was obtained from the individual(s) for the publication of any potentially identifiable images or data included in this article.

## Author contributions

MW and HZ: conceptualization, methodology, and writing—original draft preparation. HZ, XL, and SC: validation. MW: writing—review and editing. DG: visualization and funding acquisition. YZ: supervision. All authors have read and agreed to the published version of the manuscript.
